# Re-encoding of associations by recurrent plasticity increases memory capacity

**DOI:** 10.3389/fnsyn.2014.00013

**Published:** 2014-06-10

**Authors:** Daniel Medina, Christian Leibold

**Affiliations:** ^1^Department Biologie II, Ludwig-Maximilians-Universität MünchenMunich, Germany; ^2^Bernstein Center for Computational Neuroscience MunichMunich, Germany

**Keywords:** associative memory, memory capacity, sparse coding, recurrent plasticity, memory consolidation

## Abstract

Recurrent networks have been proposed as a model of associative memory. In such models, memory items are stored in the strength of connections between neurons. These modifiable connections or synapses constitute a shared resource among all stored memories, limiting the capacity of the network. Synaptic plasticity at different time scales can play an important role in optimizing the representation of associative memories, by keeping them sparse, uncorrelated and non-redundant. Here, we use a model of sequence memory to illustrate how plasticity allows a recurrent network to self-optimize by gradually re-encoding the representation of its memory items. A learning rule is used to sparsify large patterns, i.e., patterns with many active units. As a result, pattern sizes become more homogeneous, which increases the network's dynamical stability during sequence recall and allows more patterns to be stored. Last, we show that the learning rule allows for online learning in that it keeps the network in a robust dynamical steady state while storing new memories and overwriting old ones.

## Introduction

Memories are based on synaptically induced changes of intrinsically generated brain activity. Examples for such intrinsic activities are the recurring sequences of neuronal activity patterns in the hippocampus (Wilson and McNaughton, 1994; Nadasdy et al., 1999; Lee and Wilson, 2002; Davidson et al., 2009); see Buhry et al. (2011); Wikenheiser and Redish (2012) for review. Classically, these sequences were interpreted as replaying previous activity patterns. Meanwhile they have been found to also preplay future behavior (Diba and Buzsaki, 2007) or reverse replay past behavior (Foster and Wilson, 2006; Diba and Buzsaki, 2007). More recently, it has been shown that they even predict future behaviors (Gupta et al., 2010; Dragoi and Tonegawa, 2011, 2013; Pfeiffer and Foster, 2013). The diversity of these sequences has generated an equally diverse set of possible functional explanations, ranging from memory consolidation (Nakashiba et al., 2009; Jadhav et al., 2012) to memory deletion (Hoffman et al., 2007) and path planning (Azizi et al., 2013; Ponulak and Hopfield, 2013).

In this paper, we will specifically address one variant of the memory consolidation and deletion hypothesis, viz. whether these sequences can be used to drive a learning rule that allows for efficiently re-encoding memories and thereby solve the problem of catastrophic forgetting. The basic idea of this hypothesis is that new memories might be encoded by assemblies that are not optimally sparse and thus allow secure retrieval. A retrograde learning rule that propagates long-term depression (LTD) will be shown to be able to reduce these assemblies toward a level of sparseness, which is optimal from the retrieval point of view and, at the same time, allows the network to operate in a stable regime of online learning, in which old memories are overwritten by new ones. This learning rule operates on a time scale that is slower than the fast time scale of initial imprinting. As a result, new memories will be represented by a larger number of neurons (and synapses) than old memories, which are encoded more efficiently and will eventually be forgotten.

## Materials and methods

Here, we investigate memory consolidation and retrieval in a network which stores sequential associations of binary patterns (Nadal, 1991; Gibson and Robinson, 1992; Hirase and Recce, 1996; Leibold and Kempter, 2006; Kammerer et al., 2013). As in these previous papers, the dynamics is formulated in discrete time. The individual time steps can be biologically interpreted as the cycles of a collective network oscillation (e.g., hippocampal ripple oscillations; Maier et al., 2011). The employed network model is identical to that described in Medina and Leibold (2013) and lays particular emphasis on handling heterogeneous pattern sizes, i.e., the number of active neurons at any time may be different. Formally, this is expressed by the vector of coding ratios

(1) $$\phi =\{f_{0},f_{1},f_{2},\ldots ,f_{P}\}$$

where *M*_*k*_ = *f*_*k*_ *N* is the number of active neurons in the *k*-th binary pattern ξ_*k*_ ∈ {0, 1}^*N*^, *N* is the number of neurons in the network, and the indices *k* = 0, …, *P* represent each of the *P* + 1 patterns that are connected by the *P* pairwise directed associations. Unless mentioned otherwise, the coding ratios *f*_*k*_ are randomly drawn from a gamma distribution (to avoid negative patterns sizes) with mean coding ratio ϕ_0_ and standard deviation σ_ϕ_.

The associations between the individual patterns of the sequence ξ_0_, ξ_1_, ξ_2_, … are stored in the synaptic weight matrix, which is chosen according to a clipped Hebbian rule (Willshaw et al., 1969): a synapse from neuron *j* to *i* has weight *s*_*ij*_ = 0 only if a spike of neuron *i* never follows one of neuron *j* in any of the *P* associations, otherwise *s*_*ij*_ = 1. In addition to this Willshaw rule, we also allow for a morphological connectivity, i.e., a synapse from neuron *j* to neuron *i* only exists with probability *c*_*m*_ (Gibson and Robinson, 1992; Leibold and Kempter, 2006). This implies a second set of binary synaptic variables *w*_*ij*_, with *w*_*ij*_ = 1 if the respective synapse exists and *w*_*ij*_ = 0 otherwise. For such a learning rule and heterogeneous pattern sizes, it was shown in Medina and Leibold (2013) that the probability *c* of a potentiated synaptic connection (*s*_*ij*_ = 1) equals

(2) $$c=c_{m}\left(1-\prod_{k\;=\;1}^{P}(1-f_{k}f_{k\;-\;1})\right).$$

In this and related models, the choice of binary synapses facilitates the mathematical tractability of the theory, although, in biology, synaptic weights generally follow long-tailed distributions (Song et al., 2005). The long tail, however, allows one to subdivide synapses into weak and strong ones, which could be considered as being approximated by a noisy binary approach.

### Synaptic metaplasticity

According to Willshaw's learning rule, a synapse is in the potentiated state (*s*_*ij*_ = 1) if it connects two neurons that fire in sequence at least once. However, some neuron pairs may fire in sequence multiple times if they are part of the representation of consecutive patterns more than once. Although disregarded so far, the number of times a neuron pair fires in sequence is important since it tells us how many associations rely on this connection being potentiated. In order to conserve this information while using binary synapses, we consider synaptic meta levels with serial state transitions, a model similar to that proposed in Amit and Fusi (1994); Leibold and Kempter (2008).

**Figure 1:**
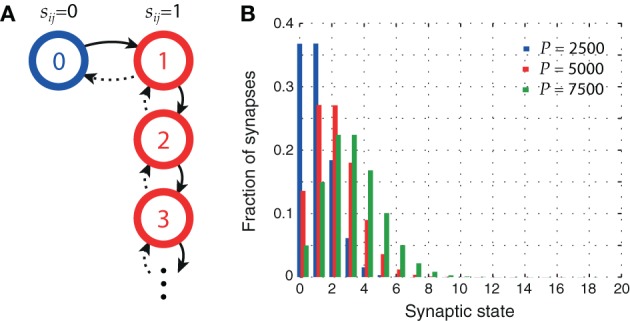
**Synaptic metaplasticity. (A)** Synaptic meta levels with serial state transitions. Learning initially potentiates the state (*solid lines*), whereas LTD signals decrement it (*dotted lines*) with probability *q*. **(B)** Distribution of synaptic states after storage of *P* patterns. The higher the network load, the more likely synapses are to be in higher meta levels. Parameters: *N* = 10^5^, *c*_*m*_ = 0.1, ϕ_0_ = 0.02, and σ_ϕ_ = 0.1 ϕ_0_.

A state diagram of our plasticity model is shown in Figure 1A. After a synapse has been potentiated once, every further occurrence of sequential firing in the sequence activation schedule increments the meta level by one, leaving the synaptic weight *s*_*ij*_ unchanged. Figure 1B shows the distribution of synaptic states in the network for three different pattern loads *P*. At higher loads, synapses are more likely to reach higher meta levels.

### Network dynamics

Following Medina and Leibold (2013), neurons are modeled using a simple threshold dynamics that translates the synaptic matrix *J*_*ij*_ = *s*_*ij*_ *w*_*ij*_ into an activity sequence: a neuron *i* fires a spike at cycle *t* + 1 if its postsynaptic potential $h_{i}(t)=\sum_{j=1}^{N}\left(w_{ij}s_{ij}-b\right)x_{j}(t)$ at time *t* exceeds the threshold θ. Here, *x*_*j*_(*t*) ∈ {0, 1} represents the binary state of neuron *j* at time *t* and *b* denotes the strength of a linear instantaneous feedback inhibition (Hirase and Recce, 1996; Kammerer et al., 2013). The negative feedback constant is chosen *b* = *c* for all subsequent simulations (Medina and Leibold, 2013).

To save computational time, most of the upcoming results are derived in a mean field approximation. To this end, in each time step, neurons are subdivided into two populations: an **On** population which is supposed to fire according to the sequence schedule and an **Off** population which is supposed to be silent (Leibold and Kempter, 2006). The number of active neurons at time step *t* can thus be divided into a number *m*_*t*_ of correctly activated neurons (hits) and a number *n*_*t*_ of incorrectly activated neurons (false alarms). Using these conventions yields the mean field dynamics (Medina and Leibold, 2013)

(3) $$\left(m_{t+1},n_{t+1}\right)=(T_{\text{On}}\left(m_{t},n_{t}\right),T_{\text{Off}}\left(m_{t},n_{t}\right))$$

with

(4) $$T_{\text{On}}(m_{t},n_{t})=M_{t+1}\Phi (\frac{\mu_{\text{On}}-b(m_{t}+n_{t})-\theta }{\sigma_{\text{On}}})$$

(5) $$T_{\text{Off}}(m_{t},n_{t})=(N-M_{t+1})\Phi (\frac{\mu_{\text{Off}}-b(m_{t}+n_{t})-\theta }{\sigma_{\text{Off}}})$$

and $\Phi (z)\equiv [1+\text{erf}(\text{z/}\sqrt{\text{2}})]\text{/2}$ denoting the cumulative distribution function of the normal distribution. Here, the mean number of synaptic inputs μ ≡ 〈*h*(*t*)〉 and the variance σ^2^ ≡ 〈*h*(*t*)^2^ 〉 − 〈*h*(*t*)〉^2^, for the **On** population, are

(6) $$\mu_{\text{On}}=c_{m}m_{t}+c_{m}\varsigma n_{t}$$

(7) $$\begin{array}{l} \sigma_{\text{On}}^{2}=c_{m}m_{t}(1-c_{m})\\ \;+c_{m}\varsigma n_{t}(1-c_{m}\varsigma +V_{\varsigma }^{2}c_{m}\varsigma (n_{t}-1)) \end{array}$$

with ς = *c*/*c*_*m*_; see Equation (2). The analog expressions for the **Off** population are

(8) $$\mu_{\text{Off}}=c_{m}\varsigma (m_{t}+n_{t})$$

(9) $$\sigma_{\text{Off}}^{2}=c_{m}\varsigma (m_{t}+n_{t})(1-c_{m}\varsigma +V_{\varsigma }^{2}c_{m}\varsigma (m_{t}+n_{t}-1)).$$

Finally, the variability coefficient *V*^2^_ς_ used in Equations (7) and (9) is given by

(10) $$V_{\varsigma }^{2}=\frac{1}{\varsigma^{2}}(2\varsigma -1+\prod_{k=1}^{P}(1-f_{k}(2f_{k-1}-f_{k-1}^{2})))-1.$$

### Retrosynaptic LTD

The replay model in Medina and Leibold (2013) assumed the synaptic matrix *J*_*ij*_ to remain constant. Synaptic plasticity may, however, take place on a slower time scale and change network dynamics between consecutive replay events. In this paper, we investigate the idea that replay evokes a retrosynaptic LTD to achieve a more efficient utilization of synaptic resources, thereby increasing storage capacity. We therefore assume that the stored patterns are initially too large and, over time, are reduced by learning such that the coding ratios *f*_*k*_ converge to an optimal value.

**Figure 2:**
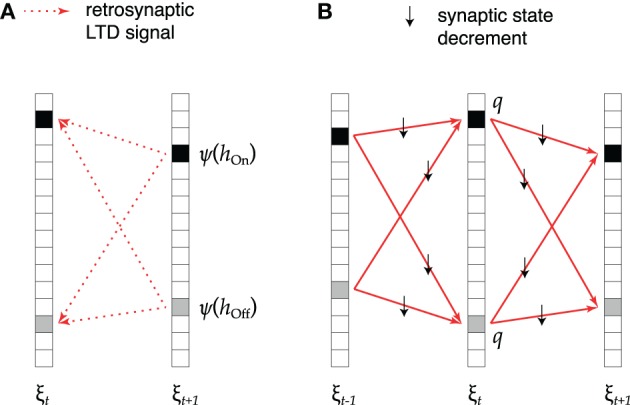
**Retrosynaptic LTD. (A)** During sequence replay, excessive depolarization *h* at time *t* + 1 triggers a retrosynaptic LTD signal that is propagated with probability ψ(*h*) to all presynaptic cells that were active at time *t* (black squares denote *hits*, gray squares denote *false alarms*). **(B)** Each cell receiving an LTD signal responds with probability *q* by decrementing the state of all its input synapses from cells that fired at time *t* − 1 and all its output synapses to cells that fired at time *t* + 1.

This idea is implemented as shown in Figure 2. During replay of association ξ_*t*_ → ξ_*t* + 1_ (Figure 2A), active cells that receive excessive synaptic input send a retrosynaptic LTD signal to all presynaptic cells which were active in the previous time step. The emission of such a signal is modeled as a stochastic process in which the emission probability ψ increases with the number *h* of synaptic inputs received by the cell like

(11) $$\psi (h)=\{\begin{array}{ll} \min(a{\left(h-h_{0}\right)}^{2},1), & h\geq h_{0},\\ 0, & \text{otherwise.} \end{array}$$

Here the parameter *h*_0_ defines a minimal pattern size *M* = *h*_0_/*c*_*m*_ beyond which plasticity signals can occur. Its choice determines the optimal memory capacity of the network, as this minimal pattern size can become a stable fixed point of the dynamics of pattern sizes.

To combine this learning rule with the mean field network dynamics, we have to find an expression for the probability *P*_*s*_ that a presynaptic cell receives at least one retrosynaptic signal. The number of inputs received at time *t* + 1 is on average μ_On_ for an **On** cell, and μ_Off_ for an **Off** cell. Thus, for an **On** cell, we have a probability (1 − ψ(μ_On_))^*c*_*m*_*m*_*t* + 1_^ of receiving no retrograde LTD signal from any active cell in the **On** population, and a probability (1 − ψ(μ_Off_))^*c_m_n*_*t* + 1_^ of receiving no retrograde LTD signal from the **Off** population. Similarly, for an **Off** cell, these probabilities are (1 − ψ(μ_On_))^*c_m_m*_*t* + 1_^ and (1 − ψ(μ_Off_))^*c_m_n*_*t* + 1_^, and thus

(12) $$P_{s}^{\text{On}}=1-{\left(1-\psi (\mu_{\text{On}})\right)}^{c_{m}m_{t+1}}{\left(1-\psi (\mu_{\text{Off}})\right)}^{c_{m}n_{t+1}}$$

(13) $$P_{s}^{\text{Off}}=1-{\left(1-\psi (\mu_{\text{On}})\right)}^{c_{m}m_{t+1}}{\left(1-\psi (\mu_{\text{Off}})\right)}^{c_{m}n_{t+1}}.$$

As illustrated in Figure 2B, upon receiving one or more LTD signals, with probability *q*, the presynaptic cell decrements *by one* the meta state of all its input synapses from the *m*_*t* − 1_ + *n*_*t* − 1_ cells that were active in the previous time step, as well as the meta state of all its output synapses to the *m*_*t* + 1_ + *n*_*t* + 1_ cells that are active in the following time step. Each synapse in the subset of decremented synapses therefore takes part in one association less. As a result, the cell no longer takes part in the neural representation of pattern ξ_*t*_, although it might still be spuriously activated during replay. On average, the size of pattern ξ_*t*_ is therefore updated according to

(14) $$M_{t}\;\to \;\Psi M_{t}$$

where $\Psi =1-q\frac{m_{t}}{M_{t}}P_{s}^{\text{On}}$.

### Online learning

If associations are stored in the network one after another (online learning), new memories will overwrite old memories (Nadal et al., 1986; Amit and Fusi, 1994), which is also known as *palimpsest* learning, and thereby the connectivity between **On** neurons of old associations is increasingly diluted. The remaining signal strength of an association *k* depends on the probability *y*_*k*_ = 1 − *p*(0|*k*) that a synapse is not in state zero, given that it participates in association ξ_*k*_ → ξ_*k* + 1_ (i.e., it connects neurons that fired in sequence during the storage of that association). To account for overwriting, the dynamical Equations (6) and (7) are modified as follows

(15) $$\mu_{\text{On}}=c_{m}y_{t}m_{t}+c_{m}\varsigma n_{t}$$

(16) $$\begin{array}{l} \sigma_{\text{On}}^{2}=c_{m}y_{t}m_{t}(1-c_{m}y_{t})\\ \;+\;c_{m}\varsigma n_{t}(1-c_{m}\varsigma +V_{\varsigma }^{2}c_{m}\varsigma (n_{t}-1)). \end{array}$$

In our model framework, the synaptic connectivity is changed in two ways. First, during imprinting of a new association, synapses increment their meta state level. Second, synaptic states are decremented via retrosynaptic LTD. To capture these changes, we define the average state distribution ρ(*s*), which describes the probability that an arbitrarily chosen synapse is in state *s*, and thus *c*/*c*_*m*_ = 1 − ρ(0).

#### Effect of synaptic potentiation on state distribution

If a new association is added that links pattern ξ_*k*_ to pattern ξ_*k* + 1_, a random synapse increases its state with probability *f*_*k*_*f*_*k* + 1_ and thus the change in the state distribution is

(17) ρ→Υρ

where

(18) $$\Upsilon =1\text{​I}+f_{k}f_{k+1}\left(\begin{array}{cccccc} -1 & \text{ ​}0 & \text{ ​}0 & & & \\ \text{ ​}1 & -1 & \text{ ​}0 & & & \\ \text{ ​}0 & \text{ ​}1 & -1 & & & \\ \text{ ​}0 & \text{ ​}0 & \text{ ​}1 & & & \\ \text{ ​}\vdots & \text{ ​}\vdots & \text{ ​}\vdots & \text{ ​}\ddots & & \\ & & & & -1 & \text{ ​}0\\ & & & & \text{ ​}1 & \text{ ​}0 \end{array}\right)$$

and 1I is the unit matrix.

#### Effect of synaptic depression on state distribution.

Conversely, retrograde LTD is described by the matrix multiplication

(19) ρ→Δρ

where

(20) $$\Delta \text{​}=\text{​}\left(\begin{array}{ccccccc} 1 & \;p_{1}+p\prime_{1} & \;p\prime_{2} & 0 & \cdots & & 0\\ 0 & \;1-p_{1}-p\prime_{1} & \;p_{2} & \;p\prime_{3} & 0 & \cdots & 0\\ \vdots & \;0 & \;1-p_{2}-p\prime_{2} & \;p_{3} & \;p\prime_{4} & 0 & \vdots \\ & \;\vdots & \;0 & \;1-p_{3}-p\prime_{3} & \;p_{4} & \;p\prime_{5} & \\ & & \;\vdots & \;0 & & & \\ & & & \;\vdots & \;\ddots & & p_{P}\\ & & & & & & 1-p_{P}-p\prime_{P} \end{array}\right)$$

and *p*_*s*_, *p*′_*s*_ are the probabilities that a replay event decreases the state *s* of a random synapse by 1 and 2, respectively.

To obtain *p*_*s*_ and *p*′_*s*_, we define the probability *p*(*s*, ↓) = ρ(*s*) *p*_*s*_ that a synapse is in state *s* and receives the signal to go down one meta level. Similarly, *p*(*s*, ↓↓) = ρ(*s*) *p*′_*s*_ is the probability that a synapse is in state *s* and receives the signal to go down two meta levels.

A depression event ↓ during replay of the association ξ_*k*_ → ξ_*k* + 1_ can have two origins: (1) the depression signal ↓^(*k*_+_)^ that is sent by a neuron of pattern ξ_*k*_ to its output synapses, and (2) the depression signal ↓^(*k*_−_)^ the neuron sends to its input synapses. Since a subpopulation of synapses may be part of both the inputs and the outputs of pattern ξ_*k*_, a synapse may be depressed twice and thus go down two levels. Since the patterns are statistically independent, both depression events are independent and thus the probability that a synapse is in state *s* and goes down by two levels upon replay of association ξ_*k*_ → ξ_*k* + 1_ is given by

(21) $$p\text{​}\left(s,↓\text{​}↓\right)=p\text{​}\left({↓}^{\left(k_{+}\right)}|s\right)p\text{​}\left({↓}^{\left(k_{-}\right)}|s\right)\rho (s).$$

Similarly, the probability *p*(*s*, −) that a synapse stays in state *s* is

(22) $$p(s,-)=\left(1-p\text{​}\left({↓}^{\left(k_{+}\right)}|s\right)\right)\left(1-p\text{​}\left({↓}^{\left(k_{-}\right)}|s\right)\right)$$

A synapse either stays in state *s*, it goes down by one state or goes down by two states, and thus

(23) ρ(s)=p(s,−)+p​(s,↓)+p​(s,↓​↓).

This normalization condition then yields the probability *p*(*s*, ↓) that a synapse is in state *s* and is decreased by one, viz.

(24) $$p\text{​}\left(s,↓\right)=p\text{​}\left(s,{↓}^{\left(k_{+}\right)}\right)+p\text{​}\left(s,{↓}^{\left(k_{-}\right)}\right)-2p\text{​}\left(s,↓\text{​}↓\right).$$

The probabilities *p*(*s*, ↓^(*k*_±_)^) can be further split up into two non-overlapping subsets of synapses, one (called *k*) that connects the **On** populations of association *k* and another one (called *k*) denoting all other synapses. Therefore we have

(25) $$p\text{​}\left(s,{↓}^{\left(k_{+}\right)}\right)=p\text{​}\left(s,{↓}^{\left(k_{+}\right)},k\right)+p\text{​}\left(s,{↓}^{\left(k_{+}\right)},\bar{k}\right)$$

(26) $$p\text{​}\left(s,{↓}^{\left(k_{-}\right)}\right)=p\text{​}\left(s,{↓}^{\left(k_{-}\right)},k-1\right)+p\text{​}\left(s,{↓}^{\left(k_{-}\right)},\bar{k-1}\right).$$

Since the LTD signal ↓ is independent of the synapse state *s*, we have

(27) $$p\text{​}\left(s,{↓}^{\left(k_{+}\right)},k\right)=p(s|k)p\text{​}\left({↓}^{\left(k_{+}\right)},k\right)$$

(28) $$p\text{​}\left(s,{↓}^{\left(k_{+}\right)},\bar{k}\right)=p(s|\bar{k})p\text{​}\left({↓}^{\left(k_{+}\right)},\bar{k}\right)$$

and in analogy for *k* − 1. The last terms on the right-hand side are obtained from equations (12) and (13) as follows

(29) $$p\text{​}\left({↓}^{\left(k_{+}\right)},k\right)=qP_{s}^{\text{On}}\frac{m_{k}m_{k+1}}{N^{2}}$$

(30) $$\begin{array}{l} p\text{​}\left({↓}^{\left(k_{+}\right)},\bar{k}\right)=qP_{s}^{\text{On}}\frac{m_{k}n_{k+1}}{N^{2}}\\ \;+qP_{s}^{\text{Off}}\frac{n_{k}\left(m_{k+1}+n_{k+1}\right)}{N^{2}} \end{array}$$

and

(31) $$p\text{​}\left({↓}^{\left(k_{-}\right)},k-1\right)=qP_{s}^{\text{On}}\frac{m_{k}m_{k-1}}{N^{2}}$$

(32) $$\begin{array}{l} p\text{​}\left({↓}^{\left(k_{-}\right)},\bar{k-1}\right)=qP_{s}^{\text{On}}\frac{m_{k}n_{k-1}}{N^{2}}\\ \;+qP_{s}^{\text{Off}}\frac{n_{k}(m_{k-1}+n_{k-1})}{N^{2}}\;. \end{array}$$

What remains to be obtained in Equations (27) and (28) are the conditional probabilities *p*(*s|k*) and *p*(*s*|*k*). From heuristic considerations, we approximate

(33) $$p(s|k)=p\text{​}\left(s-1|\bar{k}\right)r_{k}+p\text{​}\left(s|\bar{k}\right)(1-r_{k})\;.$$

Equation (33) assumes that the presence of association ξ_*k*_ → ξ_*k* + 1_ can affect the conditional state distribution *p*(*s|k*) in two ways: either it increases the state by one (*p*(*s* − 1|*k*) *r*_*k*_), or it has no effect on the state (*p*(*s*|*k*) (1 − *r*_*k*_)). The constants *r*_*k*_ can be interpreted as the fraction of synapses for which association ξ_*k*_ → ξ_*k* + 1_ contributes to the next meta level. We will refer to them as the remaining memory strength of association *k*.

Combining equation (33) with

(34) $$\rho (s)=p(s|k)p(k)+p\text{​}\left(s|\bar{k}\right)p\text{​}\left(\bar{k}\right)$$

we can recursively compute

(35) $$p(s|\bar{k})=\frac{\rho (s)-p\text{​}\left(s-1|\bar{k}\right)r_{k}f_{k}f_{k+1}}{1-r_{k}f_{k}f_{k+1}}$$

and in particular provide a connection between *r*_*k*_ and *y*_*k*_ via

(36) $$\frac{\rho (0)}{1-r_{k}f_{k}f_{k+1}}=p(0|\bar{k})=\frac{\rho (0)-f_{k}f_{k+1}(1-y_{k})}{1-f_{k}f_{k+1}}.$$

#### Effect of synaptic depression on signal connectivity

In addition to changes in the state distribution ρ that describes the *noise* connectivity during associations, retrosynaptic LTD also specifically influences the synapses between the **On** populations according to *y*_*l*_ = 1 − *p*(0|*l*). For more recent associations *y*_*l*_ will be large, whereas for older associations *y*_*l*_ will be small. The change in *y*_*l*_ that results from retrosynaptic LTD while replaying association ξ_*k*_ → ξ_*k* + 1_ is computed from the change in *p*(0|*l*),

(37) $$\begin{array}{l} p(0|l)\to p(0|l)+(p\text{​}\left(↓|1,l\right)+p\text{​}\left(↓\text{​}↓|1,l\right))p(1|l)\\ \;+p\text{​}\left(↓\text{​}↓|2,l\right)p(2|l). \end{array}$$

For associations *l* ∉ {*k* − 1, *k*} the conditional probabilities of depression are independent of the association *l*, i.e., *p*(↓|*s, l*) = *p*(↓|*s*) = *p*_*s*_ and *p*(↓↓|*s, l*) = *p*′_*s*_. The conditional state occupancies are obtained via the *r*-factors from Equation (36) as *p*(*s|l*) = *p*(*s* − 1|*l*) *r*_*l*_ + *p*(*s*|*l*) (1 − *r*_*l*_).

For associations *k* − 1 and *k*, synapses can only experience by-chance LTD from one of the two signals (association *k* − 1 from ↓^(*k*_+_)^ and association *k* from ↓^(*k*_−_)^), since LTD from the other signal would result in a decrease of the pattern size (with undiluted connectivity). Likewise there is no double decrement for these associations. As a result, the update rules for these associations are

(38) $$\begin{array}{l} p(0|k-1)\to p(0|k-1)+p\text{​}\left({↓}^{\left(k_{+}\right)}|1,k-1\right)p(1|k-1)\\ \;=p(0|k-1)+p\text{​}\left({↓}^{\left(k_{+}\right)},1|k-1\right)\\ \;=p(0|k-1)+p\text{​}\left({↓}^{\left(k_{+}\right)},1,k|k-1\right)\\ \;+p\text{​}\left({↓}^{\left(k_{+}\right)},1,\bar{k}|k-1\right)\\ \;=p(0|k-1)+p\text{​}\left({↓}^{\left(k_{+}\right)},k|k-1\right)p(1|k,k-1)\\ \;+p\text{​}\left({↓}^{\left(k_{+}\right)},\bar{k}|k-1\right)p(1|\bar{k},k-1)\\ \;=p(0|k-1)+p\text{​}\left({↓}^{\left(k_{+}\right)},k\right)p(1|k,k-1)\\ \;+p\text{​}\left({↓}^{\left(k_{+}\right)},\bar{k}\right)p(1|\bar{k},k-1) \end{array}$$

and, replacing *k* − 1 by *k*,

(39) $$\begin{array}{l} p(0|k)\to p(0|k)+p\text{​}\left({↓}^{\left(k_{-}\right)}|1,k\right)p(1|k)\\ \;=p(0|k)+p\text{​}\left({↓}^{\left(k_{-}\right)},k-1\right)p(1|k,k-1)\\ \;+p\text{​}\left({↓}^{\left(k_{-}\right)},\bar{k-1}\right)p(1|k,\bar{k-1}). \end{array}$$

The probabilities *p*(1|*k, k* − 1), *p*(1|*k*, *k* − 1) and *p*(1|*k*, *k* − 1) in Equations (38) and (39) can be obtained in analogy to

(40) $$\begin{array}{l} p(1|k,k-1)=\frac{p(1,k,k-1)}{p(k,k-1)}=\frac{p(k|1)p(k-1|1)\rho (1)}{p(k,k-1)}\\ \;=\frac{p(1|k)p(1|k-1)}{\rho (1)} \end{array}$$

due to statistical independence of the patterns.

#### Effect of synaptic depression on subthreshold variance

The dynamics of sequence replay not only depends on the mean connectivities *c* and *c*_*m*_ *y*_*k*_ but also on the second moment of the connectivity matrix as captured by *V*^2^_ς_ from Equation (10). Retrosynaptic LTD will also affect this second moment. As an approximation, we again use the *r*-factors from Equation (36), which are an estimate of the fraction of presynaptic **On** neurons that contribute to the meta level of association *k*. Thus, we can replace the coding ratio *f*_*k* − 1_ in Equations (2) and (10) by the diluted coding ratio *r*_*k* − 1_ *f*_*k* − 1_ and obtain

(41) $$V_{\varsigma }^{2}=\frac{1}{\varsigma^{2}}(2\varsigma -1+\prod_{k=1}^{P}(1-r_{k-1}f_{k-1}f_{k}(2-r_{k-1}f_{k-1})))-1.$$

Since the definition of the *r*-factors in Equation (33) implements only an approximation, the two ways of computing the mean connectivities via *c*/*c*_*m*_ = 1 − ρ(0) and *c*/*c*_*m*_ = 1 − ∏^*P*^_*k* = 1_ (1 − *f*_*k*_ *f*_*k* − 1_*r*_*k* − 1_) are slightly different. To achieve numerical robustness we obtain ρ(0) by applying Newton's method to solve the implicit Equation

(42) $$\rho (0)=\prod_{k=1}^{P}\left(1-f_{k}f_{k-1}\frac{\rho (0)-(1-y_{k})}{\rho (0)-f_{k}f_{k+1}(1-y_{k})}\right)$$

for ρ(0) in which the *r*-factors have been expressed via Equation (36).

## Results

### Retrosynaptic LTD during sequence replay sparsifies large patterns

**Figure 3:**
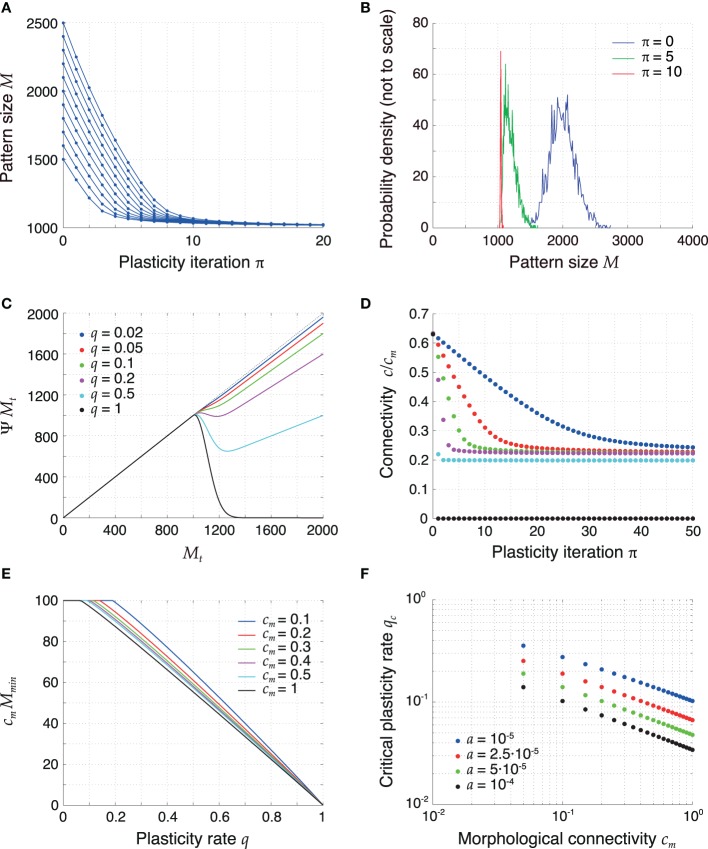
**Retrosynaptic LTD signals sparsify and homogenize the pattern size distribution. (A)** Evolution of the sizes of a few sample patterns for *q* = 0.1. **(B)** Evolution of the full pattern size distribution. Initially (*blue*), sizes are very inhomogeneous (σ_ϕ_/ϕ_0_ = 10%). After 5 iterations (*green*) of plasticity, patterns are encoded more sparsely and the sequence becomes more homogeneous. After 10 iterations (*red*), the sequence is essentially homogeneous. **(C)** The plasticity map given by (14), showing how a pattern size *M*_*t*_ is sparsified as a function of *q*. The curves shown were obtained with parameters *a* = 2.5·10^−5^ and *h*_0_ = 100 in (11) and setting *m*_*t* + 1_ = ϕ_0_ *N* and *n*_*t* + 1_ = 0 in (12). For too high *q* values (*q* ⪆ 0.2), the fixed point of the dynamics of pattern sizes at *M* = *h*_0_/*c*_*m*_ = 1000 is surpassed. **(D)** Connectivity decreases as a result of plasticity sparsifying stored patterns (*P* = 2500). The rate of decrease increases with *q*. (E) Minimal value of *c*_*m*_ Ψ *M* as a function of *q* for different values of *c*_*m*_ and constant *h*_0_. At the critical value of *q* the curves bend down in a non-differentiable way. **(F)** Critical LTD probability *q*_*c*_ as a function of *c*_*m*_ for constant *h*_0_. Other parameters: *N* = 10^5^, ϕ_0_ = 0.02, and *c*_*m*_ = 0.1 unless mentioned otherwise.

The mean field description for the pattern size changes from Equation (14) can be interpreted as a dynamical system itself, since it constitutes a discrete-time iterated map on the pattern sizes. The time scale of this dynamics is slower than the time scale of sequence replay since, during the replay of a sequence, the pattern sizes change only by a small amount. Figures 3A,B show the temporal evolution of the sizes of some example patterns and of the full distribution of pattern sizes for *q* = 0.1 that results from the mean field Equation (14). The simulations show that the pattern sizes converge to a common fixed point and, as a result, the pattern size distribution becomes delta-like. For such homogeneous pattern sizes the memory capacity is maximized (Medina and Leibold, 2013).

To more systematically analyze plasticity on the slow time scale, we revisit dynamical Equation (14) and interpret it as a one-dimensional iterated map *M*_*t*_ → Ψ *M*_*t*_. Figure 3C visualizes the iteration function Ψ *M* for different values of *q*. For small *q*, the fixed-point Equation *M* = Ψ *M* has a solution for a maximal pattern size *M* = *h*_0_/*c*_*m*_, which serves as an attractor of the discrete dynamics for all starting values *M* > *h*_0_/*c*_*m*_. If *q* is too high, the iteration function bends down for *M* > *h*_0_/*c*_*m*_ and there is no longer a single fixed point for all initial pattern sizes *M* > *h*_0_/*c*_*m*_. The critical value of *q* is thus determined by the condition

(43) $${\text{min}}_{M>h_{0}/c_{m}}(\Psi M)\leq h_{0}/c_{m}$$

which means that the minimum of the iteration function Ψ *M* for *M* > *h*_0_/*c*_*m*_ is smaller or equal *h*_0_/*c*_*m*_. The critical value *q*_*c*_ is the smallest value of *q* for which condition (43) is fulfilled and is indicated by the kink of the graphs in Figure 3E. For larger *q* the iterated map can produce pattern sizes below *h*_0_/*c*_*m*_, which are then marginally stable fix points but the resulting pattern sizes may be too small for successful replay. The critical *q*_*c*_ is not universal and depends on parameters. Most importantly, it decreases with *c*_*m*_ and *a* (Figure 3F). The critical value remained above a few percent for a wide range of parameters. Specifically, in sparsely connected networks (*c*_*m*_ ≪ 1), the choice *q* ≈ 0.05 is generally subcritical and thus allows for an optimal storage capacity.

The dynamics of pattern sizes is paralleled by a dynamics of the mean network connectivity from Equation (2); Figure 3D. A reduction of the pattern sizes leads to a corresponding decrease in connectivity. The rate of this decrease is higher for higher values of *q*. For subcritical values of *q* (0 < *q* ≤ *q*_*c*_) the average connectivity converges to a fixed point that is independent of *q*. For supercritical *q* (1 > *q* > *q*_*c*_) the connectivity converges to a lower fixed-point connectivity, indicating a substantial fraction of too small pattern sizes. In the extreme case *q* = 1 all synapses are depotentiated and the connectivity converges to 0.

### Plasticity during sequence replay increases dynamic stability

**Figure 4:**
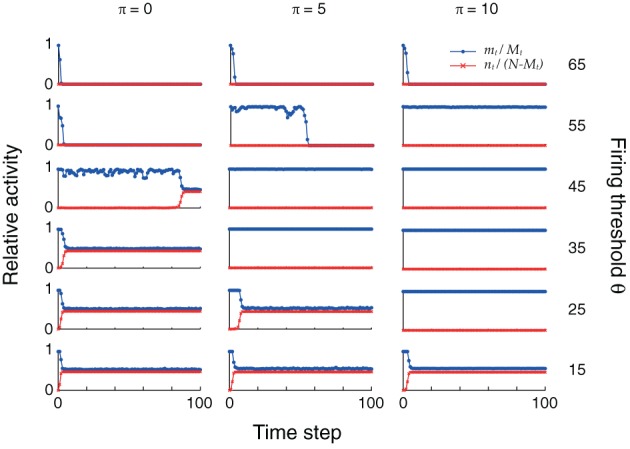
**Plasticity of pattern sizes increases the dynamic stability during sequence replay**. In all graphs, we show the fraction *m*_*t*_/*M*_*t*_ of hits (*blue*) at time step *t* and the fraction *n*_*t*_/(*N* − *M*_*t*_) of false alarms (*red*) during the replay of a sequence (only the first 100 time steps are shown), using the mean field model of Equations (3) and following. Left to right: increasing plasticity iterations. Bottom to top: increasing firing thresholds θ. The initial pattern size distribution had parameters ϕ_0_ = 0.02 and σ_ϕ_/ϕ_0_ = 10%. Other parameters were *N* = 10^5^, *c*_*m*_ = 0.1 and *P* = 2500.

The changes in connectivity due to retrosynaptic LTD are paralleled by changes of fast dynamics of sequence replay according to Equations (3) as exemplarily illustrated for three different plasticity stages (initial, after 5 and 10 iterations) and firing thresholds in Figure 4. As plasticity proceeds and the pattern size distribution in the sequence becomes more homogeneous, the activity fluctuations during replay are reduced and, eventually, allow for the whole sequence to be retrieved successfully.

In the example of Figure 4, learning extends the range of thresholds under which the network successfully replays the full sequence if the network was perfectly initialized (*m*_0_ = *M*_0_, *n*_0_ = 0). For a large threshold (e.g., θ = 55), learning allows for the emergence of ongoing sequence replay in a regime where initially no self-sustained network activity was possible. Before any plasticity takes place, the pattern sizes are highly inhomogeneous and the network falls silent almost immediately. After 5 plasticity iterations, fluctuations are reduced and the network is able to successfully retrieve more items in the sequence. Near perfect pattern retrieval (*m*_*t*_/*M*_*t*_ = 1 and *n*_*t*_/(*N* − *M*_*t*_) = 0) is made possible after 10 iterations. Similarly, for low thresholds (e.g., θ = 25), replay initially drives the network into an epileptic state (*m*_*t*_/*M*_*t*_ ≈ *n*_*t*_/(*N* − *M*_*t*_) ≈ 0.5). The reduction of pattern sizes due to learning, again, allows for ongoing sequence replay.

Defining the retrieval quality (Leibold and Kempter, 2006)

(44) $$\Gamma_{t}\equiv m_{t}/M_{t}-n_{t}/(N-M_{t})$$

as the relative difference between hit ratio and false alarm ratio, allows a better comparison of the replay performance for a large set of parameter choices. Formally this is done via the *replay success rate*, which is the fraction of runs for which at time *t* the replay quality Γ_*t*_ is above 0.5 (Medina and Leibold, 2013).

**Figure 5:**
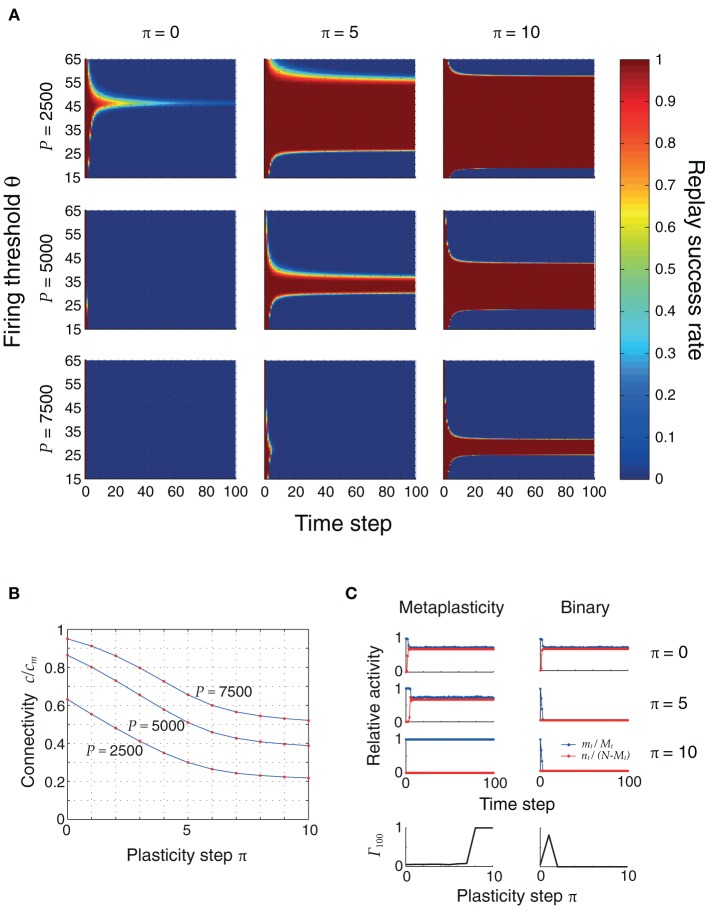
**(A)** Replay success rate over time for different firing thresholds θ and a sequence of length *Q* = 100. These plots were obtained using the mean field model, and were verified using cellular simulations. Left to right: increasing plasticity iterations. Top to bottom: increasing pattern load *P*. The initial pattern size distribution had parameters ϕ_0_ = 0.02 and σ_ϕ_/ϕ_0_ = 10%. Other parameters were: *N* = 10^5^, *c*_*m*_ = 0.1. **(B)** Connectivity decreases as the network sparsifies its stored associations. This plot was obtained by simulating the actual neural network with three different pattern loads *P* and a randomly generated coding ratio vector ϕ. The connectivity was calculated both using the mean field equation (2) (*blue*) and counting the actual number of potentiated synapses (*red dots*), showing a perfect match. **(C)** Advantage of metaplasticity (*left*) over simple binary synapses (*right*) during retrosynaptic LTD. Blue and red traces indicate hits and false alarms (as in Figure 4) for 0, 5 and 10 learning steps. The bottom row depicts the replay quality of the 100th pattern in the sequence as a function of the number of learning steps. Only with metaplasticity the replay remains stable for many learning steps.

Figure 5A shows the evolution of replay success rates for three plasticity stages and three different memory loads *P*. Initially, the pattern sizes are large and inhomogeneous, and ongoing sequence replay is not possible. Only for small loads (*P* = 2500) and for a small firing threshold range (θ ≈ 45), can the first items be retrieved with high probability. As plasticity reduces inhomogeneity and sparsifies the patterns, the range of firing thresholds θ for which the full sequence can be retrieved expands. This is made possible by a decrease in the *noise* connectivity *c*, shown in Figure 5B and verified through cellular simulations. In a modified model without synaptic meta states there was no improvement by applying repeated learning steps, since synapses were switched to an inactive state too quickly (Figure 5C).

### Online learning

**Figure 6:**
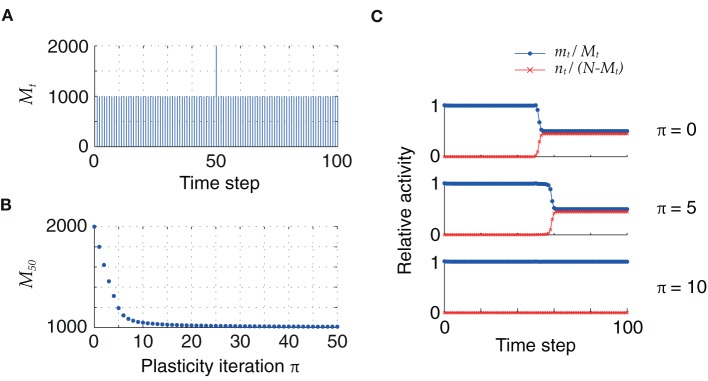
**(A)** Homogeneous sequence with a double-sized pattern at *t* = 50. **(B)** Evolution of oversized pattern with plasticity (*q* = 0.1). **(C)** Initially, sequence replay fails at *t* = 50 because of the excessive synaptic drive generated by the oversized pattern, which leads the network to an epileptic state (*top*). After 5 iterations, the network explosion is slower but still present (*middle*). Successful replay of the full sequence is possible after 10 iterations (*bottom*). Parameters: *N* = 10^5^, *c*_*m*_ = 0.1, *c* = 5%, ϕ_0_ = 0.01, θ = 26.

So far, the initial distribution of pattern sizes was centered at mean values far above the fixed point *M* = *h*_0_/*c*_*m*_. However, once the pattern size distribution has reached this optimal value, retrosynaptic LTD will only take place if a new association with an oversized pattern is added into the synaptic matrix. In our model, this can be simulated as a homogeneous sequence with one pattern of size larger than *M* = *h*_0_/*c*_*m*_, as illustrated in Figure 6: for low firing thresholds (θ = 26), the excess synaptic drive generated by an oversized pattern initially leads to sequence termination by setting the network into an epileptic state. Plasticity via retrosynaptic signals gradually reduces the size of the problematic pattern, eventually allowing for successful replay of the full sequence. This shows that retrosynaptic LTD in principle makes it possible to integrate new associations into the network, and therefore provides a possible basis for online learning, i.e., the ongoing storage of new memories.

Of course, adding new associations (increasing *P*) will consequently also increase the mean connectivity *c*, up to a point at which classically memories can no longer be retrieved (Willshaw et al., 1969; Nadal, 1991; Kammerer et al., 2013). For these large connectivities *c* the false alarms add considerable synaptic inputs such that a neuron is no longer always able to correctly decide whether it should fire or not. Using our present model of retrograde LTD, however, neurons could detect such over-excitation and may subsequently depress synapses.

To investigate whether this mechanism allows for self-organized sequence replay in a steady state, we set up a simulation in which we add sequences of 7 new patterns before each plasticity episode and monitor the retrieval quality as well as the mean connectivity. The dynamics of the connectivity *c* and *c*_*m*_ *y*_*k*_ is thereby simulated according to Section 2.4.

**Figure 7:**
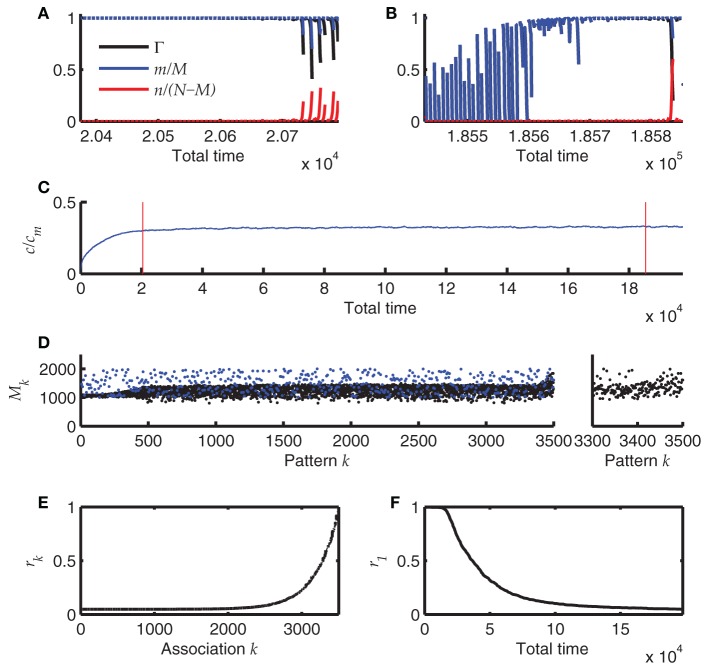
**Online learning. (A)** Replay of the 60 most recent sequences before the network has reached a steady state. Blue and red lines depict hits and false alarms, respectively, black line indicates the retrieval quality Γ. **(B)** Same as A after the network has reached its steady state. **(C)** Mean connectivity *c* as a function of time. **(D)** Pattern sizes. Blue dots indicate the sizes of the last (7th) pattern of each sequence. **(E)** The remaining memory strength *r* of all associations at the end of the simulation (500 sequences; 3500 patterns). **(F)** The memory strength of the oldest association as a function of simulation time. Parameters of the simulation were *N* = 4 · 10^4^, *c*_*m*_ = 0.1, *b* = 0.03, θ = 30, *h*_0_ = 100, *a* = 10^−5^. The simulation was terminated after having stored 500 sequences of length 7.

The result of one such simulation is summarized in Figure 7. The simulation starts with an empty network, i.e., all synapses are in state 0. Each time after storing a new sequence, the newest 60 sequences (if already available) are replayed starting with perfect initialization of the first pattern, *m* = *M* and *n* = 0. These replays induce retrosynaptic LTD. Before the network has reached a steady state, replay is generally successful for all sequences (Figure 7A) and is worse for the last recalled patterns in younger sequences, because there the pattern sizes have not yet converged to their optimum *h*_0_/*c*_*m*_ = 1000. This is because oversized patterns tend to evoke dynamical instabilities that lead to many false alarms and bad replay quality. After the network has reached a steady state, the first of the 60 replays generally fail, whereas the younger sequences can be replayed at high quality (Figure 7B). Interestingly, the mean connectivity *c* converges to its steady state more quickly than the replay dynamics (Figure 7C). The pattern sizes are slightly above their optimum *h*_0_/*c*_*m*_ (Figure 7D; note that each 7th pattern is the final pattern of each sequence and does not shrink according to the learning rule. These final patterns stay at their initial size). Only for the newest patterns the sizes reflect the initial distribution (here a uniform distribution between 1000 and 2000).

The *r* values that measure the remaining memory strength of an association (see Methods), provide an additional view on the memory capacity of the network; Figure 7E. Their convergence to zero for old memories reflects the memory time scale of the network. Additionally, the approach to the steady state is made visible if one monitors the *r* value of the oldest memory (*r*_1_) over time (Figure 7F). The convergence of *r* is much slower than the convergence of the mean connectivity *c* (Figure 7C), explaining why the replay dynamics further changes long after the mean connectivity has reached its steady state.

## Discussion

Fast hippocampal activity sequences have been hypothesized to underlie memory consolidation (Ego-Stengel and Wilson, 2010; Mölle and Born, 2011; Jadhav et al., 2012). On the cellular level, the associated re-encoding of episodic memories can either occur at the synapses between hippocampus and neocortex (Buzsaki, 1996; Frankland and Bontempi, 2005) or within the hippocampus itself. So far, hypotheses for hippocampus-intrinsic consolidation were mainly focusing on synaptic mechanisms (Frey and Morris, 1997; Milekic and Alberini, 2002; Päpper et al., 2011). The present paper provides a mechanistic model of memory re-encoding on the circuit level whereby associations between assemblies of neurons are not strengthened over time, but assemblies are reduced in size to utilize the hippocampal resources more efficiently.

Retroaxonal learning affecting both input and output synapses of a neuron has been suggested to aid stabilization of recent memories previously (Harris, 2008), although only in the context of synaptic potentiation. There, neurotrophins have been hypothesized to constitute a plausible underlying biochemical pathway. Here, we suggest a specific functional role for a retroaxonal spread of depression and have shown that it may allow a network to operate in an online mode where old memories are overwritten by new memories. Moreover, the suggested retrograde LTD predicts that depression in output synapses should be correlated with depression in input synapses.

A different mechanism suggested to reduce the overall excitatory drive in a network is synaptic scaling, whereby all synapses of an overexcited neuron undergo LTD (Turrigiano et al., 1998; Watt et al., 2000; Turrigiano, 2008; Savin et al., 2009). Retroaxonal learning is a more content-specific mechanism than synaptic scaling since it only affects synapses that have been active in the recent past and thus generally accounts for longer retention times.

Previous models of online learning (Amit and Fusi, 1994; Fusi et al., 2005; Ben Dayan Rubin and Fusi, 2007; Leibold and Kempter, 2008; Amit and Huang, 2010; Huang and Amit, 2011) usually do not explicitly take into account the network dynamics underlying the induction of plasticity. This paper presents a hypothesis of how LTD can be derived from network dynamics. The initial imprinting of the memories by LTP is still ad-hoc since we assume it to be occurring via extra-hippocampal signals.

Several other theoretical explanations for sequence replay and the sharp-wave ripple state have been suggested. (1) Sequences can be seen as avalanche-like activity patterns that are amplified by dendritic non-linearities (Memmesheimer, 2010; Jahnke et al., 2012, 2013). (2) CA1 pyramidal cell spike patterns may be triggered by strong feedforward excitation from CA3 inputs that are temporally coordinated by fast recurrent inhibition (Ylinen et al., 1995; Geisler et al., 2005; Taxidis et al., 2012). (3) The ripple oscillation may result from a network of gap-junction coupled axons (Traub et al., 1999; Traub and Bibbig, 2000; Vladimirov et al., 2013). (4) Sequences may result from a few overlapping attractor states in a recurrent network of neurons (Azizi et al., 2013). So far, these models are hardly evaluated with respect to their memory capacity (although coding capacity was probed in Azizi et al., 2013).

High memory capacities have been found in classical models of memory networks, developed independently of the hippocampal physiology, that suppose neuronal sequences to result from attractor networks with asymmetrically biased synaptic matrices (Dehaene et al., 1987; Buhmann and Schulten, 1988) in discrete time. One major drawback of these classical theories as well as the model presented here is their formulation in discrete time, which makes them hard to connect to cell-physiological properties of pyramidal cells. On the cellular level, sequence replay is most likely associated with the presence of huge precisely timed excitatory and inhibitory synaptic conductances (Maier et al., 2011). Whether and how under such conditions a neuron can fire and, more specifically, can select to fire at one specific oscillation cycle during a ripple, remains to be shown.

Sparsification of the hippocampal code may be an important intermediate step to prepare consolidation of memories in the hippocampal-neocortical loop, since generally storage capacity increases with sparseness (Nadal, 1991; Leibold and Kempter, 2006) and associating large hippocampal assemblies with neocortical states might be too costly. On the other hand, initially large assemblies might have the advantage that new associations can be retrieved more robustly. Optimal sparseness cannot be obtained by translating from one brain area to another via a random connectivity matrix, since then associations get lost as they may fall in the lower tail of the statistical distribution of the number of synaptic connections and thus do not give rise to sufficient excitation in the downstream brain area. Optimally sparse codes, hence, always require additional plasticity rules that carve out the subset of neurons that can fire reliably. The activity-driven increase in sparseness could also explain the prevalence of a few dominant preplay sequences (Dragoi and Tonegawa, 2013) that may provide an easily addressable substrate for future associations. Our model predicts that, once these sequences are connected with a memory item, the internal representation becomes more sparse and the sequences are no longer spontaneously visible. However, they are nevertheless stored within the hippocampal synaptic matrix and can be retrieved upon presentation of appropriate cue patterns.

### Conflict of interest statement

The authors declare that the research was conducted in the absence of any commercial or financial relationships that could be construed as a potential conflict of interest.
